# Antiproliferative Effects of Alkaloids from the Bulbs of *Crinum abyscinicum* Hochst. ExA. Rich

**DOI:** 10.1155/2020/2529730

**Published:** 2020-10-31

**Authors:** Besufekad Abebe, Solomon Tadesse, Ariaya Hymete, Daniel Bisrat

**Affiliations:** Department of Pharmaceutical Chemistry and Pharmacognosy, School of Pharmacy, Addis Ababa University, P.O. Box 1176, Addis Ababa, Ethiopia

## Abstract

*Crinum abyscinicum* Hochst. ExA. Rich bulb is traditionally used in Ethiopia for the treatment of various ailments including internal parasites, mastitis, rabies, colic diseases of animals, and cancer. Despite its importance in traditional cancer treatment, no research work has been reported on the antiproliferative activity of the bulb extract and its major constituents. Phytochemical investigation of the bulb extract of *C. abyscinicum* by PTLC over silica gel resulted in the isolation of two alkaloids, which were unequivocally identified as 6-hydroxycrinamine and lycorine on the basis of ^1^H- and ^13^C-NMR and MS analysis. The bulb extract, 6-hydroxycrinamine, and lycorine possessed significant antiproliferative activity, lycorine being the most active exhibiting GI_50_ values of 2.8 *μ*g/ml and 3.4 *μ*g/ml against A2780 and MV4-11 cells, respectively. Cell cycle analysis and annexin V/propidium iodide double staining in A2780 cells revealed that both compounds increased the percentage of cells in the S-phase at 30 *μ*g/ml without inducing apoptosis. Our results suggest that the antiproliferative activities of the bulb extract of *C. abyscinicum,* 6-hydroxycrinamine, and lycorine could support the traditional claim of the plant against cancer.

## 1. Introduction

Cancer is the second leading cause of death worldwide, accounting for an estimated 9.6 million deaths globally in 2018 [1]. In fact, approximately 70% of deaths from cancer occurred in low- and middle-income countries, of which Africa accounted for 7.3% of the total cancer deaths in 2018 [1]. The low cancer death reports in Africa could be due to the lack of accurate and population-based data in many of the countries [2, 3].

Most often, cancer treatment involves combination therapy, such as surgery, radiotherapy, and chemotherapy. However, high toxicity and high cost associated with mono-targeted therapies have encouraged researchers to look for alternative approaches [4]. The use of chemicals from plant extracts offers a compelling rationale for addressing the underlying biology of cancer while being efficacious, nontoxic, and cost-effective [5].

Natural products have been a rich source of medicine [6]. The huge structural diversity of natural compounds and their potential bioactivity have meant that several compounds isolated from natural sources can serve as “lead” compounds. For instance, approximately 75% of anticancer agents marketed from 1981 to 2006 were obtained or semisynthesized from plants [7]. Anticancer drugs such as vinblastine, vincristine, vinorelbine, and paclitaxel, as well as semisynthetic agents including docetaxel, camptothecin, topotecan, and irinotecan, are isolated from plants and microbes [8, 9]. The genus *Crinum* belongs to the family Amaryllidaceae and comprises approximately 130 species throughout the tropics and warm temperate regions of the world [10]. Indeed, over 500 different kinds of Amaryllidaceae alkaloids (AAs) have been found in the family Amaryllidaceae [11, 12]. Several alkaloids of the Amaryllidaceae type present in *Crinum* species are reported to exert antitumor, immunostimulating, analgesic, antiviral, antibacterial, and antifungal effects [13].

Ethiopia, like many other developing countries, heavily relies on the therapeutic benefits of traditional medicine to treat diseases [14, 15]. *C. abyscinicum* bulb powder mixed with hyena feces is applied topically for the treatment of cancer, locally called “neqersa,” in Dek Island in Lake Tana, Ethiopia [14]. Ovarian cancer is the third leading cause of cancer death among women in Ethiopia, with about 2,550 diagnosed cases and 2,000 deaths each year, while leukemia is the leading cancer incident (2,274, 10.7% of new cancer cases) in Ethiopia among men of all ages in 2018 [16, 17]. Thus, in the present study, we used A2780 and MV4-11 cells to examine the antiproliferative potential of the *C. abyscinicum* bulb extract and its two alkaloids, 6-hydroxycrinamine and lycorine. In addition, we explored the anticancer mechanisms of 6-hydroxycrinamine and lycorine by using the flow cytometer. To the best of our knowledge, no prior chemical and biological investigation has been reported on *C. abyscinicum*.

## 2. Materials and Methods

### 2.1. Collection of the Plant Material

**Figure 1:**
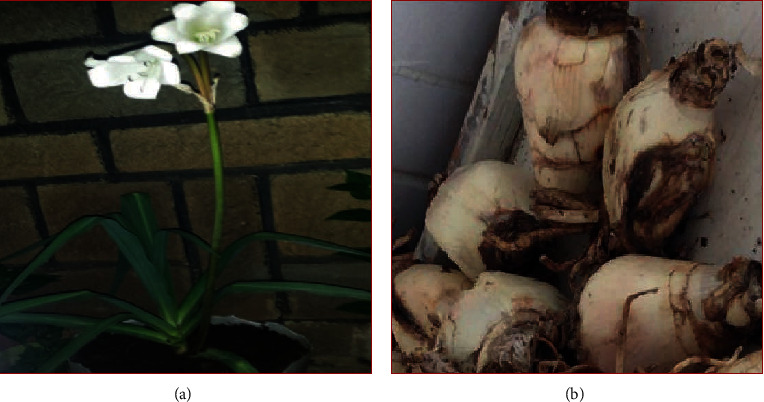
*Crinum abyscinicum*: (a) whole plant; (b) bulbs.

The bulb of *C. abyscinicum* (1 kg) (Figure 1) was collected from the town of Alelitu, 44 km northeast of Addis Ababa, in April 2015. The authenticity of the plant was confirmed by Mr. Melaku Wondafrash, Senior Botanist at the National Herbarium, Addis Ababa University (AAU), where specimens were deposited with collection number BA0001.

### 2.2. Instruments

High-resolution mass spectra acquisition was performed with an AB SCIEX Triple TOF 5600^+^ mass spectrometer (Concord, ON, Canada) in the positive ion mode using the following parameters: source temperature was set at 450°C with a curtain gas flow of 25 L/min (GS1 and GS2 both 50), the ion spray voltage was set at 4500 V, declustering potential was 50 V, the collision energy was 10 V, and the mass range was set at *m/z* 50–1500. High‐purity nitrogen gas was used for the nebulizer/Duospray™ and curtain gases. MS data acquired were processed using Analyst® TF software. Prior to MS, the sample was dissolved in methanol (1 mg/ml) and used for all sample injections. ^1^H- and ^13^C-NMR spectra were obtained using a Bruker Avance III HD spectrometer (Faellanden, Switzerland) at 500 and 125 MHz, respectively. The 2D experiments carried out included heteronuclear multiple-quantum correlation (HMQC) and heteronuclear multiple-bond correlation (HMBC). Chemical shifts are reported in units of *δ* (ppm), and coupling constants (*J*) are expressed in Hz. Multiplicity of ^1^H-NMR signals is reported as *s* = singlet, *d* = doublet, *t* = triplet, *q* = quartet, *dd* = doublet of doublets, and *m* = multiplet.

### 2.3. Extraction and Isolation of Compounds

**Figure 2:**
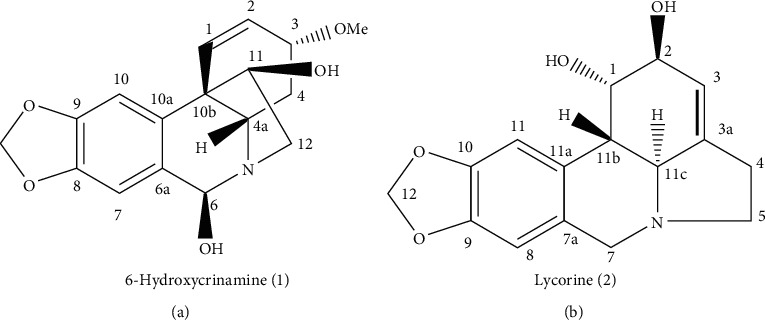
Structural formulae of alkaloids isolated from the bulbs of *Crinum abyscinicum*. (a) 6-Hydroxycrinamine (1). (b) Lycorine (2).

Dried powdered bulbs of *C. abyscinicum* (200 g) were macerated in 80% methanol (3 × 2 L, 72 h each). Then, the solution was filtered, concentrated under reduced pressure using a rota evaporator, and finally dried by a freeze drier to yield 16.3 g extract. Analytical thin-layer chromatography (TLC) was used to select a solvent system with a better resolution of the constituents of the crude drug as well as to monitor the purity of the isolated compounds. Consequently, from series of solvent systems, butanol : acetic acid : water (4 : 1 : 5) was selected as a mobile phase. The solvent was prepared by dissolving butanol, water, and then adding acetic acid. Because the solvent forms two phases, the upper layer was taken and used as a solvent system for the chromatographic separation. The extract was initially dissolved in methanol and directly applied to PTLC plates over silica gel 60 F_254_ plates (aluminium plate, 200 *μ*m, Merck KGaA, Darmstadt, Germany), and the separation was achieved using a mobile phase used for analytical chromatography. After the development, the bands were visualized under UV light at 254 and 366 nm. Two bands named BCA-1 and BCA-2 were separately scrapped off and washed with a mixture of ethyl acetate and methanol (1 : 1), filtered, and concentrated to offer two alkaloids (Figure 2) with the *R*_*f*_ value of 0.62 BCA-1 ((6-hydroxycrinamine) (1), 12 mg) and (0.44 BCA-2 (lycorine) (2), 25 mg).

### 2.4. Cell Lines and Culture Conditions

MV4-11 (human acute myeloid leukemia; American Type Culture Collection (ATCC)) and A2780 (ovarian cancer; European Collection of Authenticated Cell Cultures (ECACC)) cells were obtained from the cell bank at the Centre for Drug Discovery and Development, University of South Australia. The cell lines were maintained following ATCC recommendations either in RPMI-1640 (Roswell Park Memorial Institute), DMEM (Dulbecco's Modified Eagle's Medium), or MEM (Minimum Essential Media) with 10% fetal bovine serum. All cell lines were cultured at 37°C in a humidified incubator in the presence of 5% CO_2_. All cells were mycoplasma-tested.

### 2.5. Antiproliferative Assay

3-(4,5-Dimethylthiazol-2-yl)-2,5-diphenyltetrazolium bromide (MTT) assay was carried out on A2780 cell lines as described elsewhere [18]. In brief, 1 × 10^5^ cells/well were seeded into 96-well plates and incubated overnight at 37°C. Test samples were dissolved in dimethyl sulfoxide (DMSO), and a 3-fold dilution series was prepared in 100 *μ*L of the cell medium, added to cells (in duplicates), and incubated for 72 h at 37°C. MTT was made up as a stock of 5 mg/mL in the cell medium, and the solution was filter-sterilized. Medium was removed from cells followed by washing with 200 *μ*L/well phosphate-buffered saline. MTT solution was then added at 20 *μ*L/well and incubated in the dark at 37°C for 4 h. MTT solution was removed, and cells were again washed with 200 *μ*L of PBS. MTT dye was solubilized with 200 *μ*L/well of DMSO with agitation. Absorbance was read at 540 nm using an EnVision multilabel plate reader (PerkinElmer, Beaconsfield, Buckinghamshire, UK).

Resazurin assay was done on MV4-11 cell lines as described previously [19]. In short, cells were seeded at 5 × 10^3^ cells/well into 96-well plates and incubated overnight at 37°C, 5% CO_2_. Tested samples were diluted from 10 mM stock solution to prepare a threefold dilution series in 100 mL of the cell medium, added to cells (in duplicates), and incubated at the corresponding time point at 37°C, 5% CO_2_. Resazurin was made up as a stock of 0.1 mg/mL in the cell medium, and the solution was filter-sterilized. The resazurin solution was then added at 20 *μ*L/well and incubated in the dark at 37°C, 5% CO_2_, for 4 h. The plate was left at room temperature for 10–15 min, and absorbance was measured at 585 nm using an EnVision multilabel plate reader (PerkinElmer, Beaconsfield, Buckinghamshire, UK). The concentration of the bulb extract of *C. abyscinicum* and isolated compounds required to inhibit 50% of cell growth (GI_50_) was calculated using nonlinear regression analysis.

### 2.6. Cell Cycle Analysis

Cell cycle analysis was performed as described elsewhere [20]. Cells were seeded at 8 × 10^4^ cells per well using a 6-well plate and incubated overnight at 37°C, 5% CO_2_. After treatment with each compound, the cells were incubated for 24 h. Cells were transferred to fluorescence-activated cell sorting (FACS) tubes and centrifuged at 300 g for 5 min. Cell pellets were collected and resuspended in 1 mL of phosphate-buffered saline (PBS) and centrifuged at 300 g for 5 min. Supernatant PBS was removed, and cell pellets were fixed by adding 500 *μ*L ice-cold 70% ethanol dropwise on ice for 15 min and collected again after being centrifuged at 300 g for 5 min. Supernatant ethanol was removed, and collected pellets were incubated with propidium iodide cell cycle solution in PBS (50 *μ*g/mL propidium iodide, 0.1 mg/mL RNase A, and 0.05% Triton X-100) at room temperature for 1.5 h and analyzed with a Gallios flow cytometer (Beckman Coulter, Brea, CA, USA). Data were analyzed using Kaluza v1.2 (Beckman Coulter, Brea, CA, USA).

### 2.7. Apoptosis Assay

Apoptotic induction test was performed as described previously [20]. Cells were seeded at 8 × 10^4^ cells per well using a 6-well plate and incubated overnight at 37°C, 5% CO_2_. After treatment with each compound, the cells were incubated for 24 h. Cells were transferred to FACS tubes and centrifuged at 300 g for 5 min. Cell pellets were collected and resuspended in 1 mL of warm PBS and centrifuged at 300 g for 5 min. Supernatant PBS was removed, and cell pellets were diluted to 1 × 10^5^ cells/mL with warm PBS and centrifuged at 300 g for 5 min. Supernatant PBS was removed, and cell pellets were resuspended with 1 mL of ice-cold PBS and centrifuged at 300 g for 5 min. Supernatant PBS was removed, and cell pellets were resuspended with 100 *μ*L of 1 × binding buffer. Then, 3 *μ*L of annexin V and 3 *μ*L of propidium iodide were added to each sample with slight vortexing, and cells were incubated in the dark for 15 min. After incubation, 200 *μ*L of 1 × binding buffer was added to each sample and analyzed by the Gallios flow cytometer (Beckman Coulter, Brea, CA, USA). Data were analyzed using Kaluza v1.2 (Beckman Coulter, Brea, CA, USA).

### 2.8. Statistical Analysis

All experiments were performed in triplicate and repeated at least twice and were given as mean ± SD; representative data were selected for generating figures.

## 3. Results

### 3.1. Characterization of Isolated Compounds

Phytochemical investigation of the 80% methanol extract of *C. abyscinicum* bulbs by PTLC afforded two compounds, with *R*_*f*_ values of 0.62 (compound 1) and 0.44 (compound 2) (mobile phase: *n*-BuOH/H_2_O/AcOH/4 : 5 : 1). Compounds 1 and 2 produced orange colour when sprayed with Dragendorff's reagent (a solution of potassium bismuth iodide prepared from basic bismuth nitrate (Bi(NO_3_)_3_)), tartaric acid, and potassium iodide (KI), suggesting that they are alkaloids [21].

Compound 1 was isolated as a pale green colored amorphous solid with a pseudo-molecular ion at *m/z* = 318.1619 [M + H]^+^ in the positive-mode HR-TOF-ESI-MS (Figure S1), indicating a molecular formula of C_17_H_19_NO_5_ for compound 1. A close analysis of the spectral data of compound 1 revealed that its ^1^H- (Figure S2) and ^13^C-NMR spectra (Figures S3 and S4) were identical with those of previously reported for 6-hydroxycrinamine (see in the following), a compound isolated from the ethanolic extract of *Crinum bulbispermum* [22]. It was also reported to be present in the leaves of *Crinum yemense* [23]. Furthermore, the structure of compound 1 (Figure 2) was further confirmed by 2D-NMR, particularly by the long-range couplings between C and H observed in HMBC.

Compound 2 was obtained as a yellow colored amorphous solid. A molecular formula of C_16_H_17_NO_4_ for compound 2 was deduced from the positive-mode HR-TOF-ESI-MS (*m/z*: 288.1176 [M + H]^+^) (Figure S5). Based on its 1D (^1^H and ^13^C) (Figures S6–S8) and 2D (HMBC and HMQC) spectral data, compound 2 was unambiguously characterized as lycorine (Figure 2) [22, 24, 25]. Lycorine is the first and the most abundant alkaloid reported from the genus and the family Amaryllidaceae [26, 27].

#### 3.1.1. Spectral Data for Compounds 1 and 2

6-Hydroxycrinamine (BCA-1) (1): a pale green colored amorphous solid; *R*_*f*_ = 0.62 (*n*-BuOH/H_2_O/AcOH-4:5 : 1); HR-TOF-ESI-MS (+ve mode, Figure S1) *m/z*: 318.1619 [M + H]^+^ (exact calcd. 318.1341 for [M + H]^+^) indicating a molecular formula of C_17_H_19_NO_5_; ^1^H-NMR (500 MHz, MeOD, Figure S2) *δ*: 2.35 (2H, *m*, H-4), 3.16 (2H, *dd*, H-12), 3.45 (3H, *s*, O-Me), 3.52 (1H, *m*, H-4a), 3.88 (1H, *m*, H-11), 4.09 (1H, *m*, H-3), 5.50 (1H, *s*, H-6), 5.92 (2H, *s*, O-CH_2_-O), 6.11 (1H, *d*, H-2), 6.31 (1H, *d*, H-1), 6.78 (1H, *s*, H-10), 6.86 (1H, *s*, H-7); ^13^C-NMR (125 MHz, MeOD, Figures S3 and S4) *δ*: 29.03 (C-4), 50.41 (C-10b), 54.40 (O-CH_3_), 57.56 (C-12), 59.96 (C-4a), 76.49 (C-3), 78.23 (C-11), 87.57 (C-6), 101.03 (O-CH_2_-O), 102.36 (C-10), 108.89 (C-7), 124.38 (C-2), 127.89 (C-6a), 132.94 (C-1), 137.16 (C-10a), 146.30 (C-8), 147.88 (C-9).

Lycorine (BCA-2) (2): a yellow colored amorphous solid; *R*_*f*_ = 0.44 (*n*-BuOH/H_2_O/AcOH-4 : 5 : 1); HR-TOF-ESI MS (+ve mode, Figure S5) *m/z*: 288.1176 [M + H]^+^ (exact calcd. 288.1236 [M + H]^+^) indicating a molecular formula of C_16_H_17_NO_4_; ^1^H-NMR (500 MHz, MeOD, Figure S6) *δ*: 2.38 (1H, *m*, H-5b), 2.46 (2H, *m*, H-4), 2.62 (1H, *m*, H-11b), 2.71 (1H, *brs*, H-11c), 2.90 (1H, *d*, H‐5a), 3.54 (1H, *d*, H-7b), 4.13 (1H, *d*, H-7a), 4.19 (1H, *s*, H-2), 4.48 (1H, *s*, H-1), 5.56 (1H, *s*, H-3), 5.93 (2H, *s*, O-CH_2_-O), 6.66 (1H, *s*, H-11), 6.90 (1H, *s*, H-8); ^13^C-NMR (125 MHz, MeOD, Figures S7 and S8) *δ*: 27.91 (C-4), 39.96 (C-11b), 53.30 (C-7), 56.42 (C-5), 61.05 (C-11c), 70.56 (C-1), 71.74 (C-2), 100.89 (O-CH_2_-O), 104.63 (C-11), 106.81 (C-8), 117.76 (C-3), 128.36 (C-11a), 128.99 (C-7a), 142.31 (C-3a), 146.29 (C-10), 146.77 (C-9).

### 3.2. Antiproliferative Activity

To evaluate the antiproliferative activity of the bulbs of *C. abyscinicum*, its 80% methanol extract was tested against A2780 and MV4-11 cell lines using MTT and resazurine assays, respectively. The results indicated that the bulb extract exerted antiproliferative effect against MV4-11 (GI_50_ = 8.3 *μ*g/ml) and A2780 (GI_50_ = 20.8 *μ*g/ml) cell lines. Thus, the bulb extract of *C. abyscinicum* possesses a promising anticancer potential. In the United States National Cancer Institute plant screening program, a crude extract is generally considered to have *in vitr*o cytotoxic activity if the IC_50_ value following incubation between 48 and 72 h is less than 30 *μ*g/mL [28].

Due to the promising antiproliferative activity of the *C. abyscinicum* bulb extract and in an attempt to identify its active ingredient, we determined the antiproliferative effects of both 6-hydroxycrinamine and lycorine against the same cancer cell lines. The results are summarized in Table 1. 6-Hydroxycrinamine is active against A2780 (GI_50_ = 2.9 *μ*g/ml) and MV4-11 (GI_50_ = 5.3 *μ*g/ml) cells. Lycorine showed better activity against both A2780 (GI_50_ = 2.8 *μ*g/ml) and MV4-11 (GI_50_ = 3.4 *μ*g/ml) cells when compared with the bulb extract and 6-hydroxycrinamine (Table 1).

### 3.3. Effects on Cell Cycle and Induction of Apoptosis

**Figure 3:**
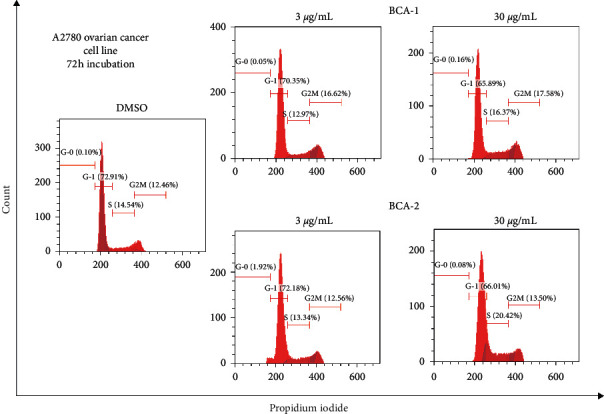
6-Hydroxycrinamine (BCA-1) causes G2/M-phase arrest at 3 *μ*g/ml and lycorine (BCA-2) causes S-phase arrest at 30 *μ*g/ml on the cell cycle of A2780 cells.

A2780 cell line, being more sensitive to both compounds (6-hydroxycrinamine and lycorine), was selected for further mechanistic studies to determine whether the growth inhibitory activity of both compounds was related to cell cycle arrest and/or induction of apoptosis. 6-Hydroxycrinamine (3 *μ*g/ml) resulted in the increase in the percentage of A2780 cells in the G2/M-phase from 12.46% to 16.62%, accompanied by a corresponding decrease in the G1- and S-phase in A2780 cells (Figure 3), whereas at higher doses (30 *μ*g/ml), lycorine increased the S-phase cell count from 14.54% to 20.42%. The observed cell cycle effects of the compounds are similar to the effects of cisplatin on A2780 cells [29].

**Figure 4:**
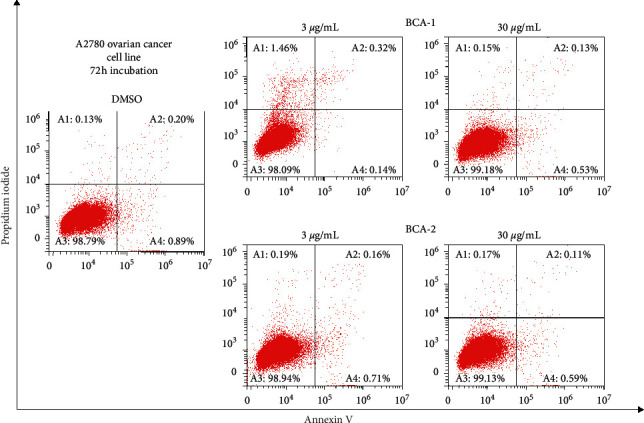
Effects of 6-hydroxycrinamine (BCA-1) and lycorine (BCA-2) on the induction of apoptosis. A1: necrotic cells, A2: cell in late apoptosis, A3: viable cells, and A4: cells at early apoptosis. BCA-1 and BCA-2 did not induce apoptosis at 3 *μ*g/ml and 30 *μ*g/ml on A2780 cells.

To determine whether apoptosis contributes to the observed antiproliferative effects of 6-hydroxycrinamine and lycorine, annexin V/propidium iodide double staining of A2780 was used. Both 6-hydroxycrinamine and lycorine did not induce apoptosis (Figure 4).

## 4. Discussion

Several studies have demonstrated that the genus *Crinum* belonging to the Amaryllidaceae family continue to yield alkaloids having interesting biological activities [13, 30]. For example, galanthamine is used for symptomatic treatment of Alzheimer disease [31], crinine and 6‐ethoxybuphandrine exhibited anticancer effect [32], and cripowellin A, B, C, and D have antiplasmodial activity [33]. In the present study, 6-hydroxycrinamine and lycorine have been found in the bulb extract of *C. abyscinicum*, a plant which is important in Ethiopian traditional medicine [14]. It is noted that lycorine showed better activity against both A2780 (GI_50_ = 2.8 *μ*g/ml) and MV4-11 (GI_50_ = 3.4 *μ*g/ml) cells when compared with the bulb extract (GI_50_ = 8.3 *μ*g/ml, MV4-11, and GI_50_ = 20.8 *μ*g/ml, A2780) and 6-hydroxycrinamine (GI_50_ = 2.9 *μ*g/ml, A2780, and GI_50_ = 5.3 *μ*g/ml, MV4-11) cells.

Lycorine has been reported to possess *in vitro* anticancer activities against ovarian carcinoma cell line (SK-OV-3) with an IC_50_ value of 0.86 *μ*g/ml [31]. Lycorine has also displayed inhibitory properties towards various cancer cell lines including lymphoma, multiple myeloma, melanoma, leukemia, lung cancer, esophageal cancer, and human anaplastic oligodendroglioma [31].

6-Hydroxycrinamine was also reported to be cytotoxic against human pancreatic (PANC1, IC_50_ = 7.20 *μ*g/ml) and prostate (DU145, IC_50_ = 2.95 *μ*g/ml) cancer cells [32]. Structurally similar compounds to 6-hydroxycrinamine such as haemanthamine and haemanthidine were also found to exert antiproliferative activity against similar cell line A2780 with a lower IC_50_ value of 215.78 *μ*g/ml and 452.01 *μ*g/ml, respectively [13], while crinamine which lacks 6-hydroxy substitute showed anticancer activity against HPV-positive cervical carcinoma SiHa (IC_50_ = 7.09 *μ*g/ml) and HPV-negative cervical carcinoma C33a (IC50 = 18.35 *μ*g/ml) cells as well as induced apoptosis without causing DNA damage [33].

Cell cycle analysis and annexin V/propidium iodide double staining in A2780 cells revealed that both compounds increased the percentage of cells in the S-phase at 30 *μ*g/ml without inducing apoptosis. The observed cell cycle effects of both compounds are similar to the effects of cisplatin on A2780 cells [29]. A2780 cells exposed to cisplatin accumulated in the G2/M-phase at 0.3 *μ*g/ml and 0.6 *μ*g/ml drug concentrations. Concomitant decreases in S- and G1-phase populations were also observed, but higher concentrations increased the relative distribution of cells in the S-phase. These events in the S- and G2/M-phase were associated with checkpoint kinases (chk1 and chk2)activation, and resultant phosphorylation and proteosomal degradation of cell division cycle 25 A (Cdc25 A). Both 6-hydroxycrinamine and lycorine did not induce apoptosis. Thus, it is possible that other mechanisms could be involved in the antiproliferative effect of the compounds. In some drug-resistant ovarian cancer cell lines such as Hey1B, antiproliferative activity was associated with induction of cytostatic effects through increasing rigidity of the actin cytoskeleton [34, 35]. However, we were unable to determine the effect of the compounds on nonmalignant cells due to their unavailability in our laboratory at the time of the study. We therefore strongly recommend further studies aiming at determining the selectivity index of 6-hydroxycrinamine and lycorine.

## 5. Conclusions

In this study, two alkaloids, 6-hydroxycrinamine and lycorine, were isolated from *C. abyscinicum*, and their anticancer activities were evaluated. Promising antiproliferative activity was established for 6-hydroxycrinamine and lycorine against A2780 epithelial ovarian cancer and MV4-11 acute myeloid leukemia cell lines. 6-Hydroxycrinamine and lycorine promote cell cycle arrest in A2780 cells without inducing apoptosis.

## Figures and Tables

**Table 1:** Antiproliferative activity of the bulb extract of *Crinum abyscinicum*, 6-hydroxycrinamine, and lycorine against A2780 and MV4-11 cell lines.

Sample name	GI_50_ (*μ*g/ml ± SD)	
	A2780	MV4-11
Bulb extract	20.8 ± 0.4	8.3 ± 0.3
6-Hydroxycrinamine (1)	2.9 ± 0.8	5.3 ± 0.5
Lycorine (2)	2.8 ± 0.1	3.4 ± 0.3
Palbociclib	0.03 ± 0.008	0.06 ± 0.002

## Data Availability

All the data used to support the findings of this study are included within the article and in the supplementary information files.

## Supplementary Materials

Supplementary Materials Figure S1: +ve mode-HR-TOF-ESI-mass spectrum of 6-hydroxycrinamine (1). Figure S2: ^1^H-NMR spectrum of 6-hydroxycrinamine (1). Figure S3: ^13^C-NMR spectrum of 6-hydroxycrinamine (1). Figure S4: DEPT-135 spectrum of 6-hydroxycrinamine (1). Figure S5: +ve mode-HR-TOF-ESI-mass spectrum of lycorine (2). Figure S6: ^1^H-NMR spectrum of lycorine (2). Figure S7: ^13^C-NMR spectrum of lycorine (2). Figure S8: DEPT-135 spectrum of lycorine (2).
