# Supplementary material for: Antiproliferative Effects of Alkaloids from the Bulbs of Crinum abyscinicum Hochst. ExA. Rich
Source: Evid Based Complement Alternat Med. 2020 Oct 31;2020:2529730. doi: 10.1155/2020/2529730 (PMC7648683; doi:10.1155/2020/2529730)
Supplement: Supplementary Materials — Figure S1: +ve mode-HR-TOF-ESI-mass spectrum of 6-hydroxycrinamine (1). Figure S2: 1H-NMR spectrum of 6-hydroxycrinamine (1). Figure S3: 13C-NMR spectrum of 6-hydroxycrinamine (1). Figure S4: DEPT-135 spectrum of 6-hydroxycrinamine (1). Figure S5: +ve mode-HR-TOF-ESI-mass spectrum of lycorine (2). Figure S6: 1H-NMR spectrum of lycorine (2). Figure S7: 13C-NMR spectrum of lycorine (2). Figure S8: DEPT-135 spectrum of lycorine (2). [file 2529730.f1.docx]

**Antiproliferative Effects of Alkaloids from the Bulbs of *Crinum abyscinicum* Hochst. ExA. Rich**

**Besufekad Abebe, Solomon Tadesse, Ariaya Hymete, Daniel Bisrat^*^**

*Department of Pharmaceutical Chemistry and Pharmacognosy, School of Pharmacy, Addis Ababa University, P.O. Box 1176, Addis Ababa, Ethiopia*

**^*^Corresponding Author**

E-mail addresses:

BA: besufekad.gebreyes@gmail.com

ST: solomon.tadesse@aau.edu.et; https://orcid.org/0000-0002-9966-2236

AH: hymete@yahoo.com

DB: daniel.bisrat@aau.edu.et; https://orcid.org/0000-0002-4007-6443


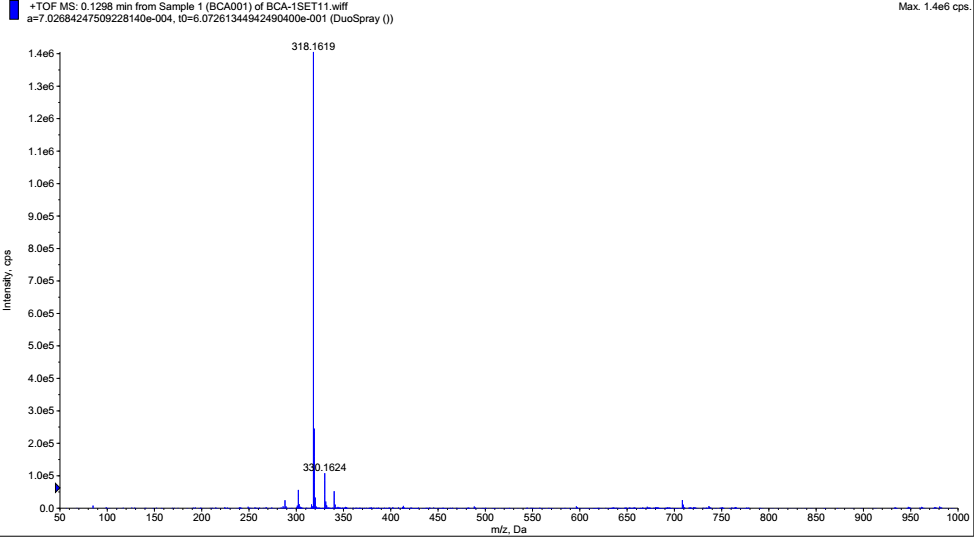


**Figure S1** (+ve mode) HR-TOF-ESI-Mass spectrum of 6-hydroxycrinamine (**1**).


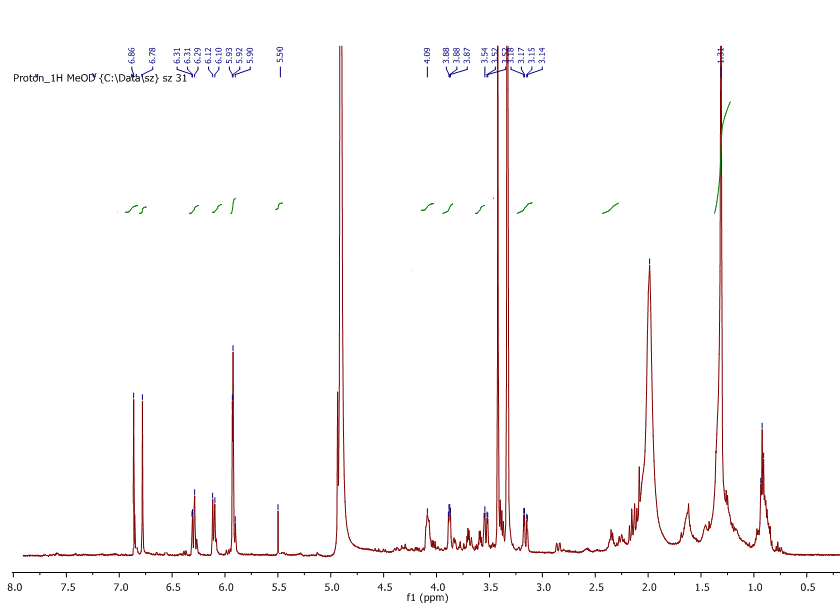


**Figure S2** ^1^H NMR spectrum of 6-hydroxycrinamine (**1**).

**
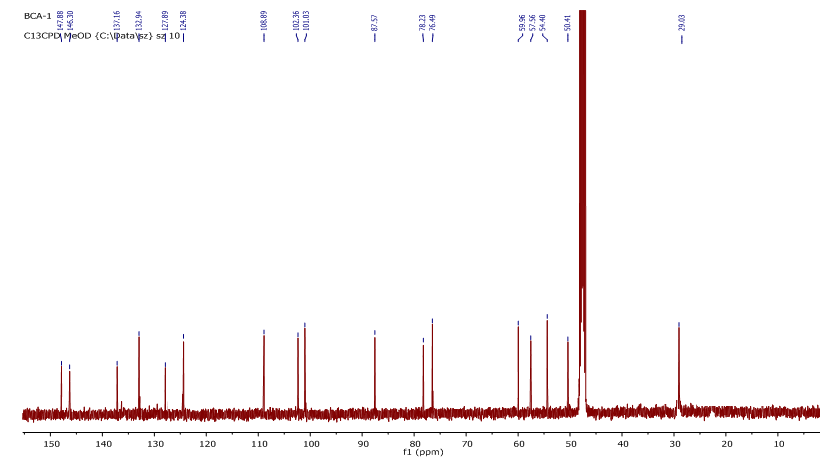
**

**Figure S3** ^13^C NMR spectrum of 6-hydroxycrinamine (**1**).


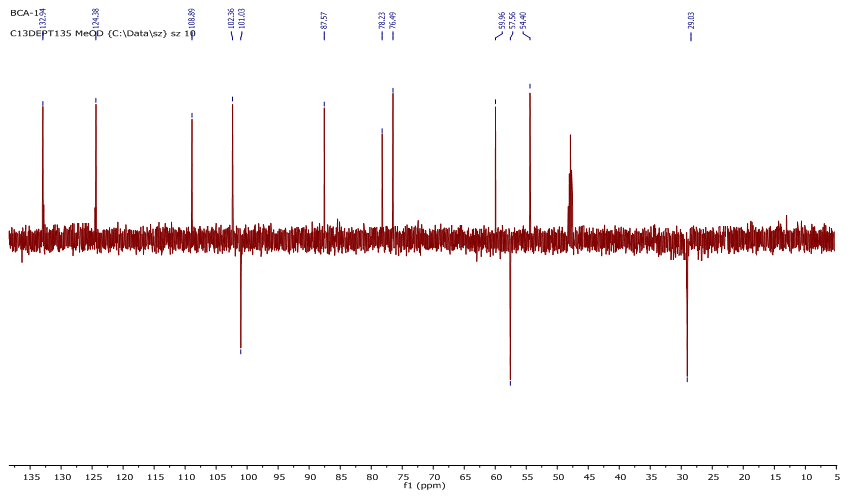


**Figure S4** DEPT-135 spectrum of 6-hydroxycrinamine (**1**).


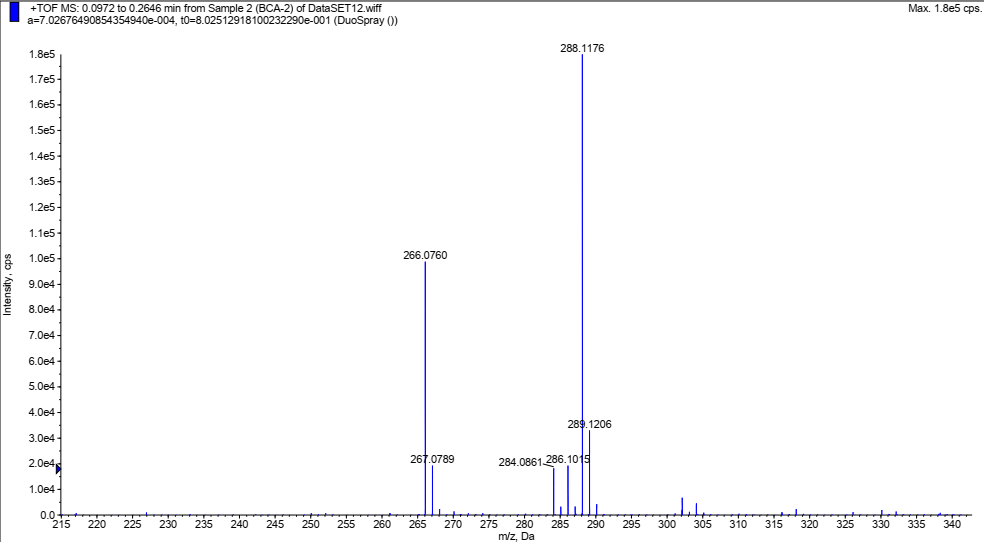


**Figure S5** (+ve mode) HR-TOF-ESI-Mass spectrum of lycorine (**2**).


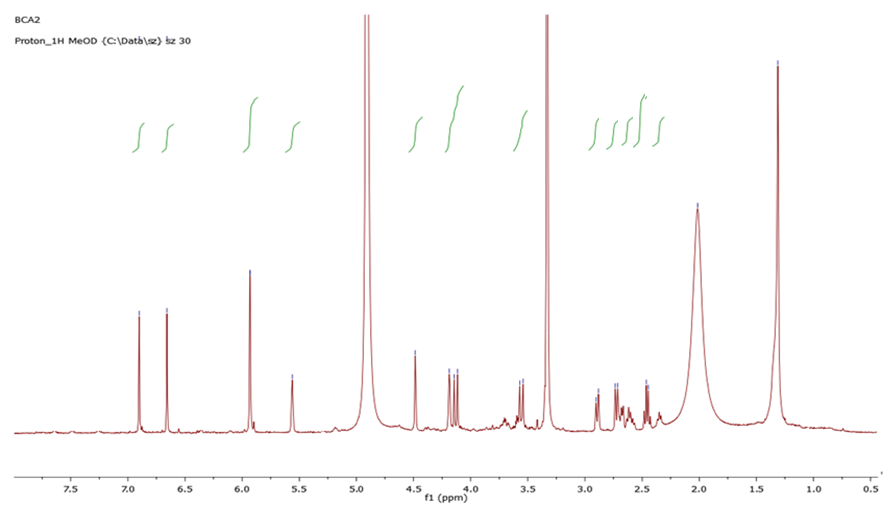


**Figure S6** ^1^H NMR spectrum of lycorine (**2**).


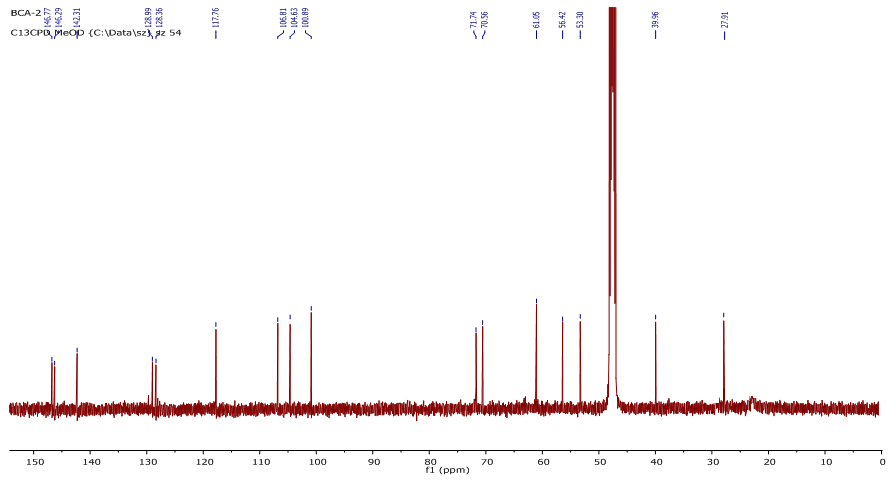


**Figure S7** ^13^C NMR spectrum of lycorine (**2**).


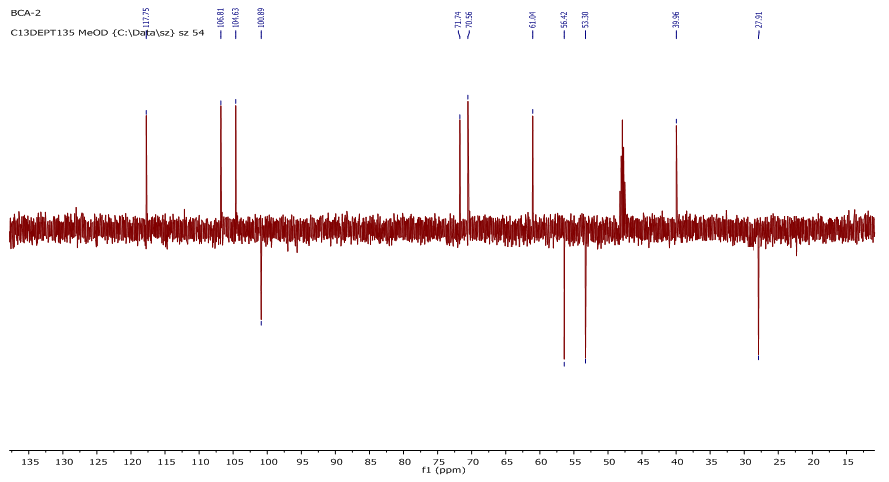


**Figure S8** DEPT-135 spectrum of lycorine (**2**).
